# Product Processing Quality Classification Model for Small-Sample and Imbalanced Data Environment

**DOI:** 10.1155/2022/9024165

**Published:** 2022-03-24

**Authors:** Feixiang Liu, Yiru Dai

**Affiliations:** Computer Integrated Manufacturing System Research Center College of Electronics and Information Engineering, Tongji University, Shanghai 201804, China

## Abstract

With the rapid development of machine learning technology, how to use machine learning technology to empower the manufacturing industry has become a research hotspot. In order to solve the problem of product quality classification in a small sample data and imbalanced data environment, this paper proposes a data generation model called MSMOTE-GAN, which is based on Mahalanobis Synthetic Minority Oversampling Technology (MSMOTE) and Generative Adversarial Network (GAN). Among them, MSMOTE is proposed to solve the problem of the sample biased to the majority class expanded by methods such as GAN in a sample imbalanced environment. Based on the traditional SMOTE method, the sample distance measurement method is modified from Euclidean distance to Mahalanobis distance, taking into account the correlation between attributes and the influence of dimensions on the sample distance. In the data generation model, MSMOTE is used to balance the positive and negative samples in the data. GAN generates fake data with the same distribution as the original data based on a balanced data set and expands the sample size to solve the problems of overfitting and insufficient model expression ability that occur when the sample size is too small. The quality classification framework of water heater liner based on the data generation model and Random Forest is constructed, and the process of the quality classification of water heater liner under the environment of small sample data and imbalanced data is fully described. This paper compares the MSMOTE-GAN model, Bootstrap, and tableGAN on the water heater liner production line data set and the public data set. The experimental result shows that the expanded data set of the MSMOTE-GAN model can effectively improve the performance of the classification model.

## 1. Introduction

In recent years, with the rapid development of machine learning technology, how to use machine learning technology to empower all walks of life has become a research hotspot. At present, many scholars are trying to combine big data analysis or machine learning technology to try to solve the problems in their research field [1], such as the combination of manufacturing and machine learning technology for anomaly detection, the combination of financial industry and big data for customer churn prediction, medicine and big data that are combined for disease diagnosis, and machine learning methods that are used to predict the results of RNA hybridization [2]. This article mainly discusses the problem of empowering manufacturing with machine learning technology. A large number of researches on machine learning empowered manufacturing have emerged, forming machine learning-based manufacturing application scenarios such as fault diagnosis, equipment health management, product surface defect detection, quality prediction, quality classification, and demand prediction. However, these application scenarios require companies to provide a large amount of data support. For companies with a higher degree of digitization, they have a large amount of data accumulation to meet the needs of machine learning algorithms. However, there are still many manufacturing companies in the early stages of digital transformation. The degree of digitalization is low, which is manifested by the insufficient number of sensor installations and lack of a large amount of data accumulation. This leads to the problem of small sample data when applying machine learning models. For machine learning models, if there is not enough data for training, the model will be overfitted, and the performance of the model will be greatly reduced. In addition, for manufacturing companies, it is more difficult to obtain abnormal data. Collecting abnormal data often means downtime or serious quality problems. Therefore, the scale of abnormal data accumulated by manufacturing companies will be smaller, so that the problem of samples imbalance will appear, but the training data with basically balanced positive and negative samples is the performance guarantee of the machine learning algorithm. The small sample data and sample imbalance mentioned above restrict the application of machine learning technology in the manufacturing industry. The data in this paper comes from a water heater liner manufacturer. At present, the product quality management of this company is still a difficult problem. The product quality screening mainly relies on manual sampling, and product quality problems cannot be found in time, which will greatly affect the production efficiency. Due to the low level of enterprise digitization, the amount of accumulated data is limited, fewer abnormal data, and the positive and negative samples are not balanced. How to classify product quality in such a data environment is a difficult problem. To achieve classification, we must first overcome the problems caused by the small sample data and imbalanced data environment.

For the problem of small sample data and sample imbalance, there have been many studies, such as random oversampling, random undersampling, SMOTE [3], and Borderline-SMOTE [4], and a series of improved SMOTE methods have been proposed to solve the problem of sample imbalance. Some scholars have proposed methods based on GAN, AE, Bootstrap, and transfer learning to solve the small sample data problem. However, when processing manufacturing data, there are still the following shortcomings.

1. The manufacturing data is highly correlated, which means that there is a relationship between attributes. At present, most of research is aiming to improve the quality of the synthesized sample from the perspective of the rationality of the minority distribution, and using the Euclidean distance to measure the sample distance. The relationship between the attributes will affect the calculation of the sample distance, causing the distance to be too large or too small, which cannot reflect the true distance value.
2. When using methods such as GAN and AE to expand small sample data, if the sample has an imbalance problem, the expanded data will be biased towards the majority, which exacerbates the problem of sample imbalance.

In order to solve the problems of lagging quality management of the water heater liner, insufficient sample data, and imbalanced samples, this paper proposes a data generation model based on MSMOTE and GAN. Except this, a product quality classification framework is proposed. The main contributions of this paper are as follows.

1. For the characteristics of strong correlation between manufacturing data attributes, the sample distance measurement method in the traditional SMOTE is modified from Euclidean distance to Mahalanobis distance in this paper, which considering the correlation between attributes and the influence of dimensions on sample distance to improve the quality of the generated samples.
2. A data generation model combining MSMOTE and GAN is proposed in this paper. The MSMOTE method is used to balance the positive and negative samples in the data, and GAN generates fake data with the same distribution as the original data based on a balanced data set and expands the sample size to solve the problems of overfitting and insufficient model expression ability when the sample size is too small.
3. The quality classification framework of water heater liner based on data generation model and Random Forest classifier is constructed. This framework completely describes the process of how to predict the processing quality of the water heater liner under a small sample data and imbalanced data environment.

The rest of this article is organized as follows.  Section 2 is the research progress of small sample data and imbalance problems.  Section 3 is an introduction to methods, including improved oversampling, data generation models, and classification frameworks. In Section 4, we conduct experiments to verify the performance of the method proposed in this paper. Finally, the conclusion and prospect are given in Section 5.

## 2. Related Work

### 2.1. Small Sample Data

Currently, there are two main methods to solve small sample data, transfer learning and data synthesis.

#### 2.1.1. Transfer Learning

Transfer learning mainly solves the problem of less sample data and poor model effect in the target domain. It transfers the model trained from the domain containing a large number of data sources to the target domain containing small sample data and uses the previous model to learn knowledge and solve problems in new domain. The source domain should be related to the target domain to a certain extent. The stronger the correlation, the better the effect of transfer learning.

Zhao [5] aimed at the problem of limited data sets in practical work and proposed a deep convolutional neural network based on transfer learning to solve the problem of small sample data sets. Wang et al. [6] proposed TL-MPM to improve the accuracy of model classification in a small sample data environment. Xiao et al. [7] combined the improved TrAdaBoost and convolutional neural network to improve the accuracy of fault diagnosis. Xia et al. [8] proposed a transfer learning method based on LSTM to explore the similarities between diseases in response to the small number of clinical key data samples. Cao et al. [9] proposed a stacked autoencoding neural network based on weighted mixed nuclear migration component analysis to solve the problem of insufficient numbers for rolling bearing state recognition. Elene et al. [10] proposed an automatic detection method of COVID-19 infection based on chest X-ray images, using transfer learning to solve the problem of insufficient X-ray image data of patients with COVID-19, and achieved good classification results. But they did not consider the problem of sample imbalance.

The above studies have adopted improved transfer learning methods to solve the problem of small sample data, but there are some drawbacks when using the transfer learning method, which are difficult to avoid. It is necessary to find numerous data sets close to the target field, and the use conditions are more stringent. The problem of the water heater liner manufacturing enterprise to be solved in this article is that it is very difficult to find data sets in similar fields due to the complicated process involved. Therefore, a small sample data processing method based on data synthesis is used in this paper.

#### 2.1.2. Data Synthesis

Data synthesis refers to the extension of training data by learning the data distribution of the original small sample data and generating new samples distributed with the original data by machine learning model. The methods used in data synthesis include GAN, AE, Reinforcement learning, SMOTE, and Bootstrap.

At present, the data generation models based on GAN and AE mostly focus on image, audio, or text data, due to rich information contained in such data. But the structured table data represents a single content, which needs to be combined with the distribution information of the whole sample to carry out research. In recent years, there have been many studies using GAN to generate image data, and few articles use GAN and AE to generate structured table data.

Han et al. [11] proposed a method for generating unlabeled samples based on a recurrent GAN in response to the problem of fully supervised learning requiring a large amount of labelled image data and facing the problem of data imbalance. The labelled samples are collectively used as the training set to better explore the discriminative relationship between positive and negative samples. Liu et al. [12] studied the relationship between fuzzy images and semantic segmentation images for the problem of the difficulty in extracting and expressing the texture of fog images and proposed a GAN for fuzzy image semantic segmentation to synthesize semantic segmentation images. Li et al. [13] considered using GAN to expand the WOA13 data set, which mainly solved the problem of small sample data and did not encounter the problem of sample imbalance. Experiments show that the expanded data set of the GAN can effectively improve the accuracy of ResNet. But too much data will not continue to improve the accuracy of the model. Zhao et al. [14] proposed an Enhanced Laplacian Pyramid GAN, based on the Laplacian pyramid to capture the high-frequency details of the image. Zhang [15] studied how to generate RGB images from sketches and proposed a color image conversion method for face sketches based on GAN and edge optimization. GAN can effectively improve the effect of image translation models.

The above-mentioned GAN model and its improvement methods are used to expand or generate image data, but this paper is to expand structured data through generative models. There are also some related researches.

Xu et al. [16] put forward Tabular GAN (TGAN) model in 2018, which uses GAN to generate tabular data and implicitly learn the probability distribution of data set. In 2019 [17], on the basis of TGAN, the author proposes a Conditional Tabular GAN (CTGAN) based on Conditional GAN. The experiment shows that CTGAN is better than Bayesian in generating tabular data. Park [18] and Song [19] proposed a tableGAN, which solved the problem that traditional GAN could not be used for structured data table generation. The former used the model to protect privacy, and the latter used the model to expand the classification model training data set and improve the classification model accuracy. Mahmud et al. [20] and Zhao [21] proposed a fault diagnosis method based on variational AE and convolutional neural network to solve the problems of few fault samples and imbalanced data in the fault diagnosis method of the above-mentioned drive. Carden [22] proposed a strategy learning method, which uses part of the researchers' knowledge of probability transfer structure to transform it into an approximate generation model, from which to generate synthetic data.

In summary, TGAN, CTGAN, and tableGAN only solve the problem of sample expansion but do not solve the problem of low data authenticity and single category caused by imbalanced training data. In order to generate high quality data samples, the problem of sample imbalance needs to be solved firstly.

### 2.2. Imbalanced Data

At present, there are two main methods to solve the problem of sample imbalance, which are from the data perspective and the algorithm perspective. Since solving the problem of sample imbalance from an algorithm perspective requires numerous samples, so we focus on solving the problem of sample imbalance from a data perspective. From the data perspective, it is to change the distribution between the data to turn the imbalanced data into balanced data through the sampling method. The common methods can be divided into two categories according to the principle, the minority type oversampling and the majority type undersampling. There are some important research results in undersampling, such as random undersampling, which randomly deletes part of the samples in the majority class to make the samples more balanced.

Laurikkala [23] proposed the Neighborhood Cleaning Rule based on the random undersampling method. Tsai et al. [24] introduced cluster analysis and instance selection on the basis of random undersampling and proposed a new undersampling method. Ponce et al. [25] proposed a two-stage undersampling technique that combines the DBSCAN clustering algorithm and the minimum spanning tree algorithm to remove noise samples. Zhang et al. [26] paid more attention to data overlap when studying the problem of imbalanced data and proposed a Random Forest Cleaning Rule, which is an undersampling algorithm to remove samples with a given boundary threshold across domains. Kang et al. [27] proposed a new undersampling method in response to the problem that noise in minority samples will reduce the performance of the classifier. A noise filter is added before resampling. The indicators have been significantly improved, and the authors further explored the relationship between algorithm performance and imbalance rate.

Whether it is random undersampling or improved undersampling methods combined with clustering algorithms, there is a problem. Undersampling will eliminate data in most classes, which may cause some important information losing, especially for small sample data. The sample size itself is not large, and if part of it is removed, subsequent machine learning models will have overfitting problems.

Oversampling increases the number of minority samples by generating new data or resampling the original data, so that the samples tend to be balanced. Random oversampling is simply repeated sampling from a minority of samples. However, the sample repetition rate is too high and does not contribute to subsequent model training.

In order to solve this problem, Chawla [3] proposed an oversampling method that synthesizes new data based on the original data distribution, namely, SMOTE. This method can synthesize new minority data while keeping all the majority data, so that the sample number tends to be balanced, which reduces the possibility of overfitting and improves the generalization performance of the model. With the deepening of research, some improved methods of SMOTE have also been proposed.

Han et al. [4] proposed Borderline-SMOTE, which used KNN to classify minority samples into three categories, safety, danger, and noise, and then oversampled dangerous minority samples to add boundary samples for distinguishing categories. He et al. [28] proposed an adaptive weighted distribution of synthetic sampling method ADASYN. The idea of this algorithm is that the number of samples synthesized by the minority samples with different weights is different, and the more difficult minority classes are synthesized with more data. Douzas et al. [29] proposed the G-SMOTE algorithm, which synthesizes minority samples in the geometric region around each selected minority sample. Luo et al. [30] proposed the use of imbalanced triangles to synthesize data based on SMOTE linear interpolation. Yang et al. [31] proposed a clustering oversampling method combining SMOTE and FINCH in order to solve the problem of large sample noise in current oversampling methods and determined the sample synthesis scheme for each minority class according to the clustering sparsity. K-means SMOTE algorithm uses K-means to cluster the input data set and performs SMOTE oversampling in clusters with a large number of samples in the minority class to avoid the generation of noise [32]. The FCMSMT algorithm combines fuzzy c-mean (FCM) with SMOTE, clusters minority classes, oversampling samples of minority classes with few samples, and reduces errors within and between classes [33]. Huo [34] proposed an improved minority sample synthesis oversampling technique based on genetic algorithm to solve the problem that the same sampling rate for different minority samples affects the performance of the algorithm, improving the accuracy of the classification algorithm for imbalanced data sets. As we all know, improving the recognition accuracy of identifying minority classes is a crucial problem faced by classification models in an imbalanced sample environment. Effective feature selection methods can help improve the accuracy of minority class recognition. Liu et al. [35] proposed a method based on weighting. The embedded feature selection method of damping coefficient is compared with the feature selection methods such as Chi2, F-statistic, and Gini index. The method proposed by the author has outstanding performance.

In summary, the use of undersampling to balance samples requires the elimination of most types of data, which may lose key information. Using random oversampling to expand the minority samples will result in a high sample repetition rate and has no application value. The deep learning model learns the sample distribution to expand the samples of the minority class. This method faces the problem that the generated samples tend to the majority class when there are only a few minorities class samples. SMOTE and its improved method balance the samples by synthesizing new samples considering the distribution of samples, increase the diversity of minority samples, and reduce the possibility of model overfitting. This shows that the SMOTE method is more suitable for dealing with the sample imbalance problem in small sample data environment compared to undersampling, random oversampling, and deep learning generative models.

In response to the above problems, this paper proposed a data generation model that combined MSMOTE and GAN to solve the problem of small sample data expansion under imbalanced samples and improve the performance of the classification model.

### 2.3. Product Quality Classification

Product quality classification refers to the use of machine learning classification model to mine the nonlinear coupling relationship between product production process parameters and product quality, so as to facilitate the subsequent prediction of product quality according to process parameters and solve the problem of lagging quality management of water heater liner manufacturers.

Wang et al. [36] proposed a product quality classification mining method based on BP neural network to mine the relationship between process parameters and final acetone quality classification in acetone refining process. Wang et al. [37] discussed the quality analysis of a new tapping machine. The correlation between the quality of the nut and the tapping process was analyzed by using various regression trees and learning methods. Karaali [38] proposed to use convolution neural network model for multiclassification of marble quality. Manimala [39] proposed a data selection method based on fuzzy c-means clustering for power quality event classification. Sankhye et al. [40] used supervised machine learning methods such as classification to predict product compliance quality using manufacturing data collected during production.

Another problem that this paper studies is the quality classification model, which is a nonlinear fitting problem. Most of the above quality classification models are based on neural network models, considering the nonlinear fitting ability of neural network models. However, considering that the final result is used for quality classification, this paper uses Random Forest, which performs well in the classification model. This model can integrate the classification results of multiple weak classifiers and calculate the final category by voting, which greatly improves the classification accuracy of the model.

## 3. Materials and Methods

### 3.1. Synthetic Minority Oversampling Technique and MSMOTE

**Figure 1:**
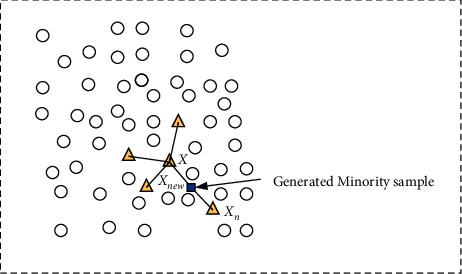
SMOTE algorithm schematic diagram.

SMOTE [3] is essentially a sample oversampling method. It is an improved scheme based on the random oversampling method. The random oversampling method only expands the minority data by simple sample resampling, and there is no new data generation, resulting in more duplicate data in the sample, which will bring overfitting problems to the model, and the training model will over adapt to the minority data. The synthetic minority class oversampling technology is proposed to solve this problem. SMOTE algorithm uses the similarity of features between minority samples to establish artificial data; it assumes that the samples between the closer minority are also minority and adds the minority samples to the data set by randomly sampling two points of the minority to do linear interpolation. The principle of the algorithm is shown in Figure 1. The specific algorithm flow is as follows:

(1) Choose a sample from the minority samples *X*.
(2) Calculate the Euclidean distance between the sample *X* and the other minority samples; get its *K* neighbors.
  (1) $$\begin{array}{c} \text{dist}\left(X,Xi\right)=\sqrt{\sum_{j=1}^{n}{\left(x_{j}\;-\;xi_{j}\right)}^{2}}. \end{array}$$
(3) According to the sample imbalance rate, the sampling ratio is set to determine the sampling rate *N*. For each minority sample *X*, a sample is randomly selected from its *K* -nearest neighbors, assuming that the selected nearest neighbor sample is *X*_*n*_.
(4) For each randomly selected nearest neighbor minority sample *X*_*n*_, the new samples are constructed according to the following formula (2) with the original sample *X*.

(2) $$\begin{array}{c} X_{\text{new}}=X+\text{rand}\left(0,1\right)*\left(X-X_{n}\right), \end{array}$$

where *X*_new_ is a newly generated sample, and rand is a random function. Then, repeat the steps (3) and (4) until the new minority class samples are balanced with the majority class samples, and the SMOTE algorithm ends.

The traditional SMOTE method uses Euclidean distance as a measure of sample distance. The most obvious disadvantage of this method is that the differences in the dimensions of the different attributes of the sample are treated equally, and the distance calculation does not consider the correlation between the different attributes of the sample. Problems with different dimensions can be solved by normalization and standardization. For the characteristics of strong correlation between manufacturing data attributes, this paper considers using Mahalanobis distance to replace Euclidean distance as a measure of sample distance, introduces covariance to measure whether there is a correlation between attributes of each dimension, and eliminates the influence of the correlation between attributes on the calculation of sample distance. This article calls the SMOTE that modifies the distance measurement method MSMOTE.

Calculating the Mahalanobis distance between the selected sample and the other minority samples, *S* is the covariance matrix. The Mahalanobis distance between the vectors *X*_*i*_ and *X*_*j*_ is defined as

(3) $$\begin{array}{c} {\text{dist}}_{M}\left(Xi,Xj\right)=\sqrt{{\left(X_{i}\;-X_{j}\right)}^{T}S^{-1}\left(X_{i}\;-X_{j}\right)}. \end{array}$$

Changing the formula (1) for calculating the sample distance in the above SMOTE algorithm steps (2) to formula (3), the rest of the steps are the same as those of the traditional SMOTE algorithm, so there is no further elaboration here.

### 3.2. Generative Adversarial Networks

GAN is a generative model first proposed by Goodfellow [41] in 2014; it has become a hot research direction in the field of artificial intelligence. The idea of GAN comes from two-person zero-sum game. GAN mainly includes two parts, namely, generator and discriminator. Through the game between generator and discriminator, the accuracy of data generated by generator and the accuracy of discriminator's classification can be improved. The generator will generate the data with the same probability distribution as the original data as far as possible, generated data, and the original data will be used as the input of the discriminator. Under the repeated training of the generator continuously generating data and the discriminator judging the true and false data, the Nash equilibrium between the generator and the discriminator is finally realized. It is precisely because the generator of GAN can generate the same distribution as the original data that GAN is often used to solve the problem of poor effect of machine learning model due to the lack of data. At present, the application of GAN is more in the field of image, and there is less research on the generation of structured data, but there are also some scholars.

**Figure 2:**
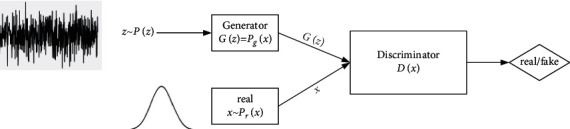
Basic structure of GAN.

The basic structure of GAN is shown in Figure 2.

The purpose of discriminator *D*(*x*, *φ*) is to distinguish the input sample *x* from the real data *P*_*r*_(*x*) or fake data generated by generator *P*_*g*_(*x*). In fact, the discriminator is a binary classification model. The label *y*=1 indicates that the data is real data, and *y*=0 indicates that the data is fake data. The output of discriminator *D*(*x*, *φ*) is the probability that *x* belongs to the real data distribution *P*_*r*_(*x*), as shown in the following formula:

(4) $$\begin{array}{c} P\left(y=1|x\right)=D\left(x,\varphi \right). \end{array}$$

The probability that the sample comes from the generator is shown in the following formula:

(5) $$\begin{array}{c} P\left(y=0|x\right)=1-D\left(x,\varphi \right). \end{array}$$

Given a sample (*x*, *y*), *y*={1,0} means that it comes from *P*_*r*_(*x*) or *P*_*g*_(*x*). The objective function of the discriminator is to minimize the Cross Entropy Loss, as shown in the following formula:

(6) $$\begin{array}{c} \underset{\varphi }{\min}\left(E_{x}\left[y\;\log\;P\left(y=\left(1|x\right)\right)+\left(1-y\right)\log\;P\left(y=\left(0|x\right)\right)\right]\right). \end{array}$$

Suppose that distribution *P*(*x*) is determined by distribution *P*_*r*_(*x*) and distribution *P*_*g*_(*x*). In other words, *P*(*x*)=(1/2)(*P*_*r*_(*x*)+*P*_*g*_(*x*)), and then the above formula is equivalent to the following formula:

(7) $$\begin{array}{c} \underset{\varphi }{\max}E_{x∼P_{r}\left(x\right)}\left[\log\;D\left(x,\varphi \right)\right]+E_{z∼P\left(z\right)}\left[\log\left(1-D\left(G\left(z,\theta \right),\varphi \right)\right)\right]. \end{array}$$

Among them, *θ* and *φ* are the parameters of generator and discriminator, respectively.

The purpose of the generator is to let the discriminator judge the fake data generated by itself as true data, assuming that the original data *x* follows the true distribution *P*_*r*_(*x*), and there is a noise vector *z* which obeys the standard normal distribution *P*(*z*) in the low dimensional space. The function of the generator is to construct a mapping function through the neural network to establish the relationship of between *P*_*r*_(*x*) and *P*_*g*_(*x*), making the sample *G*(*z*) generated by the generator obey the true distribution *P*_*r*_(*x*). The objective function of the generator is as the following formula:

(8) $$\begin{array}{c} \underset{\theta }{\max}\left(E_{z∼P\left(z\right)}\left[\log\;D\left(G\left(z,\theta \right),\varphi \right)\right]\right)=\underset{\theta }{\min}\left(E_{z∼P\left(z\right)}\left[\log\left(1-D\left(G\left(z,\theta \right),\varphi \right)\right)\right]\right). \end{array}$$

The goal of the generator is to minimize the JS divergence of the generated data distribution and the real data distribution.

According to the objective function of the generator and discriminator, the objective function of GAN is as the following formula:

(9) $$\begin{array}{c} \underset{G}{\min}\left(\underset{D}{\max}V\left(D,G\right)\right)=E_{x∼P_{r}\left(x\right)}\left(\log\;D\left(x,\varphi \right)\right)+E_{z∼P\left(z\right)}\left[\log\left(1-D\left(G\left(z,\theta \right),\varphi \right)\right)\right]. \end{array}$$

In order to generate high simulation data that can deceive the discriminator, the generator and discriminator need to be trained repeatedly to optimize the network performance. First of all, we need to train the discriminator. The optimization of the discriminator is realized by maximizing *V*(*D*, *G*) which is the objective function of the discriminator mentioned above, *E*_*r*_=*E*_*x*∼*P*_*r*_(*x*)_[log  *D*(*x*, *φ*)] describes the sample *x* which obeys the real data distribution, and *P*_*r*_(*x*) is judged as real mathematical expectation by discriminator. It is correct for the discriminator to judge the true data as the true sample. Therefore, in order to improve the ability of discriminator to judge the true and fake data, we need to maximize *E*_*r*_ · *E*_*f* _=*E*_*z*∼*P*(*z*)_[log(1 − *D*(*G*(*z*, *θ*), *φ*))] describes the sample *z* which obeys the noise distribution *P*(*z*), and the data generated by generator *G*(*z*, *θ*) is judged as fake mathematical expectation by discriminator. Because the generated data is fake sample, it is necessary to maximize *E*_*f*_ and improve the probability that the discriminator will judge the real data as true and the generated data as fake. After the discriminator has a certain discriminating ability, it begins to train the generator. According to the above formula (8), the objective function of the generator is min_*θ*_*E*_*f*_=min_*θ*_(*E*_*z*∼*P*(*z*)_[log(1 − *D*(*G*(*z*, *θ*), *φ*))]). The purpose of the generator is to let the generated data deceive the discriminator, so that the output of the discriminator *D*(*G*(*z*, *θ*), *φ*) is infinitely close to 1. The purpose of the generator is to minimize the maximum value of the discriminator objective function, which represents the similarity between the real data distribution and the generated data distribution, and JS divergence is used to measure the similarity between the two distributions. The discriminator and generator are trained alternately according to the above process until the balance state is reached, and the generated data is the same as the real data.

### 3.3. Random Forest

Random Forest [42] is a classifier based on bagging and random subspace partition strategy proposed by Breiman in 2001. It is composed of multiple decision tree models, and there is no association between different decision trees. Specifically, the traditional decision tree selects an optimal attribute in the current node's attribute set based on information purity criteria, while Random Forest introduces random attribute selection in the training process of decision tree. Due to the poor classification ability of a single decision tree, each decision tree in the Random Forest is allowed to classify and judge separately during the classification task, and each decision tree will get a classification result and finally select the most likely classification after voting statistics. The general process of Random Forest is as follows.

(1) The Bootstrap resampling technique is used to select *n* samples from random samples.
(2) *K* features are randomly selected from all the features, and the decision tree is built with these features for the selected samples.
(3) Repeat the above two steps *m* times to generate *m* decision trees and form a Random Forest.
(4) The classification result of the new data is determined by the score formed by the voting number of the decision tree. The simple voting process can be expressed by the following formula:
  (10) $$\begin{array}{c} C\left(x\right)=\underset{Y}{\arg\max}\sum_{i=1}^{m}I\left(c_{i}\left(x\right)=Y\right), \end{array}$$

where *C*(*x*) is the final result of classification, *c*_*i*_(*x*) is the classification result of the ith decision tree, *Y* is the category label, and *I*(*·*) is the indicative function.

### 3.4. Data Generation Model Based on MSMOTE-GAN

**Figure 3:**
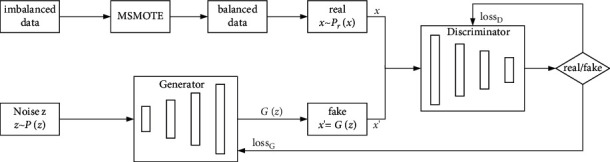
Data generation model architecture diagram.

It can be seen from the related work that TGAN, CTGAN, and tableGAN only solve the problem of sample expansion but do not really solve the problem of low authenticity and single category of generated data caused by imbalanced training data. In this paper, the MSMOTE method is proposed to solve the problem of positive and negative sample imbalance, and the data generation model is composed of the GAN to solve the problem of small sample data environment and imbalanced data. The architecture of MSMOTE-GAN data generation model is shown in Figure 3. Firstly, the imbalanced data is oversampled by MSMOTE method for a few classes, and the data set with balanced positive and negative samples is obtained. Then, randomly sample several vectors *x* from the real data set *P*_*r*_(*x*). And then, the noise vector *z* which obeys the distribution *P*(*z*) is randomly sampled as the input of the generator, and the generator generates *x*′=*G*(*z*). *x*  and *x*′ together are the input of the discriminator, and then, the generator and the discriminator according to their respective loss function, namely, loss_*D*_ and loss_*G*_, use Adam momentum optimization method to optimize the model parameters, finally achieving Nash equilibrium. The data generated by the generator cannot be distinguished from the real data by discriminator.

The specific network architecture of generator and discriminator is as follows.

#### 3.4.1. Generator Architecture

The input of the generator is the noise vector, which obeys the specific distribution. The network structure consists of four layers. The first three layers include deconvolution layer and batch normalization layer, and the activation function is ReLU. The fourth layer is the output layer and has only deconvolution layer, and the activation function is sigmoid.

#### 3.4.2. Discriminator Architecture

The input of the discriminator is the real data that obeys the specific distribution and the sample data generated by the generator. The network structure consists of four layers. The first three layers include convolution layer and batch normalization layer, and the activation function is Leaky-ReLU. The fourth layer is the output layer and has only linear layer, and the activation function is sigmoid.

#### 3.4.3. Loss Function

Loss function is an indicator of neural network performance, that is, how far the current neural network does not fit the monitoring data. The neural network model optimizes the model parameters by minimizing the loss function. Considering the data generation model proposed in this paper, the purpose of this paper is to make the data distribution *G*(*z*) and the real data distribution *P*_*r*_(*x*) generated by the generator as close as possible. The cross entropy is used as the loss function of the model, because the cross entropy is usually used to measure the difference between the two probability distributions. Generally, the smaller the cross entropy, the higher the similarity between the two probability distributions, and the direction of optimizing model parameters to improve the performance of the model is to minimize the cross entropy.

### 3.5. Quality Classification Framework of Water Heater Liner

**Figure 4:**
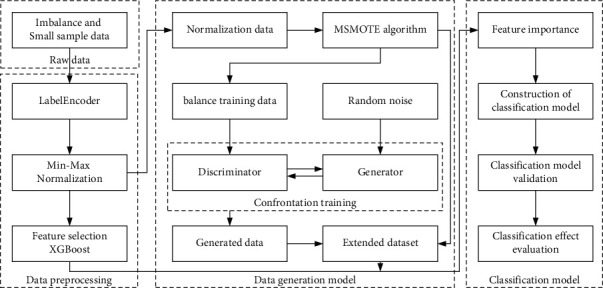
Flow chart for quality classification of water heater liner.

In order to solve the problem of classifying the product quality of water heater liner manufacturers in the environment of small sample data and sample imbalance, this paper proposes a product quality classification framework based on MSMOTE-GAN data generation model. The specific process is shown in Figure 4.

1. The process parameter data and water heater liner quality data obtained from enterprises are imported, which have the problems of imbalanced positive and negative samples and small sample data.
2. Then, the label encoding, data transformation, feature selection, and other data preprocessing are carried out. Label encoding transforms classification labels into numerical data. Data transformation transforms the attribute values of all features into the range of [0, 1] to eliminate dimensions. Feature selection is to select features that are important for subsequent quality prediction models and improve the performance of classification models.
3. The normalized data after data transformation is used as the input of MSMOTE algorithm, which is mainly used to solve the problem of poor performance of classification model caused by too few negative samples.
4. The balanced positive and negative examples are used as the input to GAN model generator, and then the generator and the discriminator are trained. Finally, the data generated by the generator cannot be distinguished by the discriminator.
5. A data generation model based on MSMOTE-GAN is used to expand the data set, which is combined with the original data set to form a joint data set.
6. According to the results of feature selection, the key factors affecting the tank quality of water heater are selected as features to construct the classification model. According to the joint data set, the training set and test set are divided to verify the classification effect of the model, and the evaluation index of the classification model is determined. According to the evaluation index, the influence of the data generation model on the performance of the classification model in the small sample data environment is evaluated.

## 4. Experimental Study

In this section, we compare the effects of origin data, SMOTE extended data, MSMOTE extended data, and MSMOTE-GAN extended data on the performance of the classification model on the water heater liner (WHL) data set firstly. Then, we compare the effects of the proposed method MSMOTE-GAN with Bootstrap and tableGAN on the performance of the classification model and conduct experiments on SPECTF, Page Blocks, and WHL data sets, respectively.

### 4.1. Problem Description

**Figure 5:**
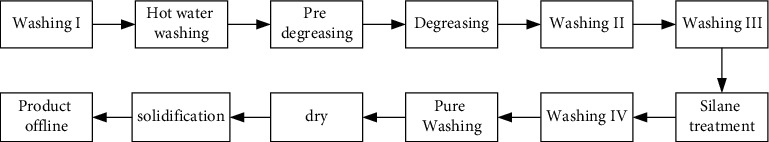
Process flow chart of water heater liner powder spraying.

The process flow chart of water heater liner powder spraying is shown in Figure 5. It needs to go through dozens of processes such as water washing, hot water washing, predegreasing, degreasing, silane treatment, drying, and curing before the final assembly. Each process involves many process parameters.

The above process involves 21 process control parameters. Any process parameter problem will lead to quality problems in the final water heater liner, which will affect the order scheduling and economic benefits of the enterprise. Therefore, if we can distinguish the product quality in advance, we can save the cost of the enterprise. The purpose of product quality classification and prediction is achieved by mining the relationship between water heater liner quality and process control parameters. However, through the actual investigation, it is found that there are few data about process control parameters and product quality inspection in enterprises at present, because most of them are manually transcribed. If we use machine learning method for data mining, we also face the problem of how to maintain the high classification accuracy of the model in the data environment of small sample data and imbalanced sample.

### 4.2. Dataset and Experimental Platform

This paper uses three data sets for model training and performance comparison. The first data set comes from the water heater liner production line, which is referred to as Water Heater Liner (WHL) in this paper. The second data set is the UCI public data set SPECTF. The third data set is the UCI public data set Page Blocks. The three data sets will be briefly introduced below.

#### 4.2.1. WHL Dataset

The WHL data set comes from a powder spraying production line for the water heater liner. It includes process parameters and product quality inspection data collected on-site. There are a total of 1133 valid data samples, including 1000 positive samples and 133 negative samples. The imbalance rate of the data set is defined as the ratio of the majority class sample to the minority class sample, and the sample imbalance rate of WHL is 7.5. In addition, the data set involves 21 process parameters during the powder spraying process of water heater liner, a label indicating whether the quality is qualified or not. The classification model needs to classify the product quality according to the 21 process parameters provided.

Parameters affecting the quality of water heater liner are shown in Table 1.

#### 4.2.2. SPECTF Dataset

The dataset describes diagnosing of cardiac Single Proton Emission Computed Tomography (SPECT) images. Each of the patients is classified into two categories: normal and abnormal. The database of 267 SPECT image sets (patients) was processed to extract features that summarize the original SPECT images. As a result, 44 continuous feature patterns were created for each patient.

#### 4.2.3. Page Blocks Dataset

The Page Blocks dataset is collected by a segmentation process, and the dataset consists in classifying all the blocks of the page layout of a document. This is an essential step in document analysis in order to separate text from graphic areas. Indeed, the five classes are as follows: text (1), horizontal line (2), graphic (3), vertical line (4), and picture (5). In this paper, we adjust the dataset to a binary classification dataset, the minority class sample is picture (5), and the remaining four classes are used as the majority class sample.

When using the above three data sets to train and test the model, we divide the data set into a training set and a test set. In view of the small amount of data in this paper, if the test set data is too small, it will aggravate the overfitting of the model, so we use the more classic 7 : 3 division ratio, 70% of the training set, and 30% of the test set. In addition, if the data distribution is not consistent during the process of dividing the data set, additional deviations will be introduced to affect the final result. Therefore, this paper will keep the imbalance rate of the training set and the test set as consistent as possible with the original data set. The division of training set and test set, the number of positive and negative cases, and the sample imbalance rate (IR) are shown in Table 2.

#### 4.2.4. Experimental Platform

The experimental environment of this paper is Intel Xeon 5238 CPU@2.1 Hz x 2, 1T SSD, Tesla T4 GPUx 4, 256G running memory, 64 bit Ubuntu 20.04 operating system. The whole model is implemented on TensorFlow platform.

### 4.3. Data Preprocessing

#### 4.3.1. Label Encoder

Because product quality inspection data and product quality category data (Label) are nonnumerical data, they need to be converted into numerical data for subsequent model construction. Product quality inspection data include appearance, thickness, adhesion, cracking resistance, hardness, impact resistance, and final product quality. Labels are expressed by P and O. In this paper, Label Encoder method is used to encode the above data. The coding result is P corresponding to number 1 and O corresponding to number 0. Among them, there are only qualified and unqualified product quality categories, and any unqualified quality inspection result is unqualified product quality.

#### 4.3.2. Min-Max Normalization

It can be seen from Table 1 that each data representing different attributes has dimensions, and there is no comparability between each feature. Therefore, it is necessary to carry out dimensionless processing on the process parameter data before building the MSMOTE-GAN model and the subsequent classification model. In this paper, Min-Max Normalization is used to make the data dimensionless. The original process parameter data is linearly transformed and mapped to [0, 1]. The specific principle is shown in the following formula:

(11) $$\begin{array}{c} v^{\prime }=\frac{v-{\text{Min}}_{a}}{{\text{Max}}_{a}-{\text{Min}}_{a}}, \end{array}$$

where *a* is an attribute of the original data, Min_*a*_ is the minimum value of the attribute, Max_*a*_ is the maximum value of the attribute, *v* is the value of the attribute, and *v*′ is the normalized value.

#### 4.3.3. Feature Selection

**Figure 6:**
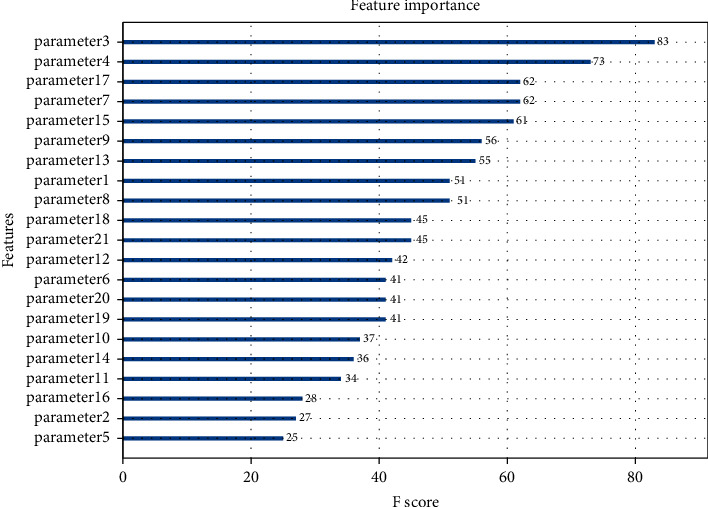
Feature importance ranking chart.

The ranking diagram of feature importance is shown in Figure 6.

It can be seen from Table 1 that there are many factors affecting product quality, but not all data attributes are closely related to product quality. If all data attributes are taken as the input variables of the model, it will not only increase the computational burden of the algorithm, increase the computational time, and even lead to inaccurate prediction results. In this paper, XGBoost algorithm is used for feature selection, and the importance score of each attribute can be directly obtained by using this method. The importance score can reflect the value of features in the model to enhance the decision tree construction. In this paper, the top 15 features are used as the features of the subsequent classification model.

### 4.4. Performance Metrics

Because of the imbalance between positive and negative samples in WHL, we cannot simply use the classification accuracy as an index to measure the performance of the model. In this paper, the confusion matrix is used to calculate the Accuracy, Precision rate, Recall rate, F1 score, and AUC value for comprehensive judgment. The specific indicators are as follows.

#### 4.4.1. Confusion Matrix

Confusion matrix structure is shown in Table 3, where TP is the number of positive samples predicted as positive samples, FN is the number of positive samples predicted as negative, FP is the number of negative samples predicted to be positive samples, and TN is the number of negative samples predicted as negative samples.

#### 4.4.2. Accuracy

Regarding the proportion of samples with correct classification to the total number of samples, the calculation formula is shown in the following formula:

(12) $$\begin{array}{c} \text{Accuracy}=\frac{\text{TP}+\text{TN}}{\text{TP}+\text{TN}+\text{FP}+\text{FN}}. \end{array}$$

#### 4.4.3. Precision

Regarding the ratio of the number of true positive samples to the number of predicted positive samples, the calculation formula is shown in the following formula:

(13) $$\begin{array}{c} \text{Precision}=\frac{\text{TP}}{\text{TP}+\text{FP}}. \end{array}$$

#### 4.4.4. Recall

Regarding the proportion of positive samples with correct classification in total samples with correct classification, the calculation formula is shown in the following formula:

(14) $$\begin{array}{c} \text{Recall}=\frac{\text{TP}}{\text{TP}+\text{FN}}. \end{array}$$

#### 4.4.5. F1 Score

Regarding the comprehensive index reflecting the Precision and Recall rate, the calculation formula is shown in the following formula:

(15) $$\begin{array}{c} F1-\text{score}=\frac{2*\text{Recall}*\text{Precision}}{\text{Precision}+\text{Recall}}. \end{array}$$

#### 4.4.6. ROC Curve and AUC Value

ROC curve and AUC value are often used as the most important evaluation index to measure the stability of the model in the model evaluation stage of binary classification problem. According to the confusion matrix, we can get another two indicators: true positive rate: TPR=TP/(TP+FN)  and false positive rate: FPR=FP/(TN+FP) . The ROC curve is obtained by taking the true positive rate (TPR) as the vertical axis and the false positive rate (FPR) as the horizontal axis, and AUC is the area under the ROC curve. The range of AUC is [0.5, 1], and 0.5 corresponds to the diagonal “random guess model.”

### 4.5. Generative Adversarial Networks Model Training Process and Parameter Setting

In this paper, Materials and Methods section has introduced the network architecture of generator and discriminator for GAN. Here, we briefly introduce the training process of GAN and some hyperparameter configurations used in this model. The specific process is as follows.

(1) The parameters of the generator are fixed, and the discriminator is trained. Only the parameters of the discriminator are updated, sampling *m* data samples *x* from the real data distribution *P*_*r*_(*x*).
(2) Sampling *m* noise vector *z* from a random noise distribution *P*(*z*).
(3) Taking the random noise vector *z* as the input of generator, *m* generated data are obtained, and *x*′=*G*(*z*, *θ*) takes *x* and *x*′ as the input of the discriminator.
(4) The loss function of the discriminator *D*(*x*, *φ*) is calculated to maximize *V*(*D*, *G*), and the smaller the *D*(*x*′, *φ*), the better. The discriminator can easily distinguish the real data from the generated data. The principle is shown in the following formula:
  (16) $$\begin{array}{c} \underset{\varphi }{\max}\left(V\left(D,G\right)=\frac{1}{m}\sum_{i=1}^{m}\log\;D\left(x^{i}\right)+\frac{1}{m}\sum_{i=1}^{m}\log\left(1-D\left(x^{\prime i}\right)\right)\right). \end{array}$$
(5) The parameters of the discriminator are updated by Adam gradient descent algorithm as the following formula:
  (17) $$\begin{array}{c} \varphi =\text{Adam}\left(\nabla \varphi \left(V\left(D,G\right)\right),\varphi \right). \end{array}$$
(6) After the parameters of the discriminator are updated *K* times, the generator is trained, and *m* vectors are sampled from a random noise distribution as the input *z*_*g*_ of the generator *G*(*z*).
(7) The loss function of generator *G*(*z*) is calculated, and minimizing *V* enables the discriminator to misjudge the generator as a real sample. The principle is shown in the following formula:
  (18) $$\begin{array}{c} V=\frac{1}{m}\sum_{i=1}^{m}\text{log}\left(1-\left(D\left(G\left(x^{\prime i}\right)\right)\right)\right.. \end{array}$$
(8) Updating generator parameters using Adam gradient descent algorithm, the principle is shown in the following formula:
  (19) $$\begin{array}{c} \theta =\text{Adam}\left(\nabla \theta \left(V\right),\theta \right). \end{array}$$

In the process of training the generator, the parameters of the generator cannot change too much and can be updated several times less. In this paper, the discriminator is set to be updated 5 times, and the generator is set to be updated 1 time to ensure that the discriminator's discrimination ability is strong enough. In addition, the batch size of the model is set to 64, the training round is set to 200, and Adam momentum optimization method is used to update the network parameters of generator and discriminator, and the learning rate is set to 0.0002. Cross entropy is used as the loss function of generator and discriminator, and cross entropy is used to measure the distance between the generated data distribution and the real data distribution. Iterative training is carried out according to the above process until Nash equilibrium is reached. The generator can generate fake data, which is similar to the real data distribution, and the discriminator cannot identify whether the data is from the real data set or the generated data. Some training parameters of the GAN model are shown in Table 4.

The training of GAN only depends on the loss function, which cannot verify the quality of the generated data, and whether the model converges or not, because the loss functions of the trained generator and discriminator of GAN should be entangled each other in the ideal state.

At present, there are mainly quantitative and qualitative methods to evaluate the quality of generated samples. Quantitative ones mean that there are some quantitative indicators, such as Inception Score (IS) [43], Mode Score [44], and Fréchet Inception Distance (FID) [45]. These indicators are used to measure the quality of the generated image, but this paper generates structured data, and there is no currently appropriate quantitative index. The quality of the generated structured data is mainly indirectly reflected by the subsequent classification model; that is, the improvement of the effect of the classification model proves that the quality of the generated samples is high. The samples generated by qualitative evaluation are mainly for image data. Users can directly observe whether the generated samples are similar to the original samples, but for structured data, the quality of the generated samples cannot be judged by observation alone. Therefore, the sample quality generated by model is indirectly evaluated by the improvement of classification model performance.

### 4.6. Experimental Results and Analysis

The comparison experiment in this paper mainly includes two parts. The first part is to compare the impact of SMOTE, MSMOTE, and MSMOTE-GAN expanded data sets on the performance of the classification model on the WHL data set. The selected classifiers include Decision Trees (DT), Random Forests (RF), Multilayer Perceptron (MLP), and XGBoost.

The second part is to further verify the adaptability of the MSMOTE-GAN model to small sample data and imbalance problem. Compare the effects of MSMOTE-GAN, Bootstrap, and tableGAN on the performance of the classification model in a small sample data and imbalanced data environment on the SPECTF, Page Blocks, and WHL data sets.

#### 4.6.1. Origin Data

The WHL data set used in this paper contains 1000 positive samples and 133 negative samples, with a sample imbalance rate of 7.5. In the test set, there are 303 positive samples and 37 negative samples. The Random Forest classification model is used to directly classify the original data. The resulting confusion matrix is shown in Table 5.

**Figure 7:**
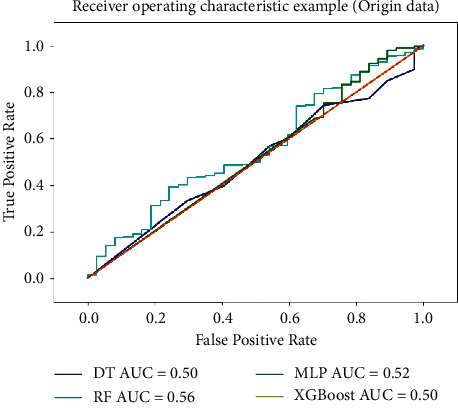
ROC curve based on original data set.

From the above confusion matrix (Table 5) and accuracy table (Table 6), it can be seen that the accuracy index of classification model training with imbalanced sample data sets has no reference value, due to positive examples account for more than 89% of all samples, and the accuracy rate of all classification models is 89.1%. From the confusion matrix, we can see that the model classifies all samples as positive examples. Therefore, this article uses the ROC curve and AUC value to measure the pros and cons of the classification model. The ROC curve of the classification model under imbalanced sample environment is shown in Figure 7.

It can be seen from Figure 7 that the AUC values of the four classification models fluctuate around 0.5. According to the standard for judging the quality of the classifier, when AUC = 0.5, the probability of the model and random guessing is the same, and the model has no predictive value. When AUC <0.5, the effect of the classification model is worse than the effect of random guessing. It can be seen from the ROC curve that the four classification models are equivalent to random guessing, and the models have no application value, and they cannot be used as the main model in the product quality classification process of manufacturing enterprises.

#### 4.6.2. Balance Data Based on SMOTE

**Figure 8:**
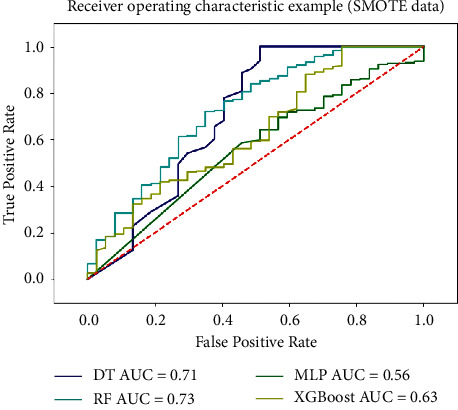
ROC curve based on balanced data set (SMOTE).

Aiming at the problem of sample imbalance, SMOTE is used to expand the minority samples. The minority samples in this paper are negative samples, with a total of 133 samples. Among them, there are 96 negative samples in the training set and 697 positive samples. The SMOTE method is used to expand the negative samples to 697, and the expanded negative samples and positive samples are used as the training set of the model. It was validated on the above four classification models, and the ROC curve is shown in Figure 8.

Compared with the results of the model before the balanced sample, the AUC values of all the classification models are improved, combined with the AUC to determine the quality of the classifier standard, 0.5 < AUC < 1, and model performance is better than random guessing and has certain application value.

#### 4.6.3. Balance Data Based on MSMOTE

**Figure 9:**
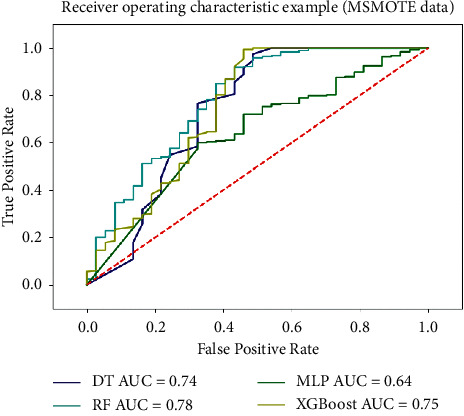
ROC curve based on balanced data set (MSMOTE).

Aiming at the problem of imbalanced manufacturing data samples, this paper proposes an MSMOTE oversampling method, in which the Mahalanobis distance replaced the classical Euclidean distance to balance the samples. The data set size and the classification model are the same. The ROC curve of the model is as shown in Figure 9. Compared with the traditional SMOTE, MSMOTE has more advantages in processing complex related manufacturing data, the AUC value of the classification model is obviously improved, and the classification accuracy of the model has been also improved.

#### 4.6.4. Extended Dataset Based on MSMTOE-GAN

**Figure 10:**
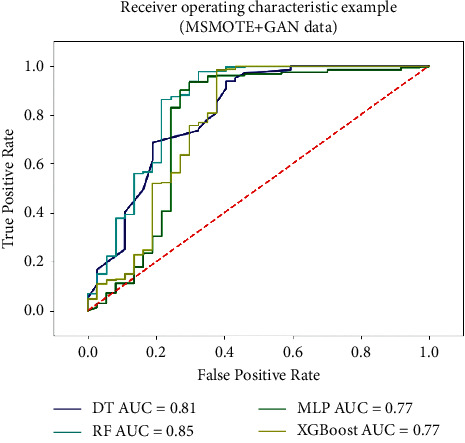
ROC curve based on extended and balanced data (MSMOTE-GAN).

It can be seen from Table 7 that although the classification performance of the model is improved compared to the data set without any processing, the accuracy of the model still needs to be improved. In view of the fact that the current number of training samples is too small, this paper proposes the MSMOTE-GAN method to balance and expand samples to provide richer feature information, thereby improving the accuracy of the classification model. Based on the balanced training set, the sample is expanded to double the original training set and combined with the original training set as a joint training set for classification model training. The test set remains unchanged. The ROC curve of each classification model and the AUC value is shown in Figure 10. It can be seen from the ROC curve that the AUC value has a certain improvement on the basis of the MSMOTE model, which proves that the data set after the expansion of the MSMOTE-GAN model can improve the classification performance of the classification model.

The Accuracy, Precision, and Recall of based on extended and balanced data are shown in Table 7. It can be seen from Table 7 that, compared to the data set after the balance of the MSMOTE method, the Accuracy, Precision, Recall, and F1 value of the four classification models have been significantly improved by using the MSMOTE-GAN model to expand the data set. It can be seen that the SMTOE-GAN based data generation model proposed in this paper can solve the problem of sample imbalance and small sample data, effectively improving the performance of the classification model. The expanded data set can improve the performance of multiple classifiers, indicating that the data generation model based on MSMOTE-GAN has strong model applicability.

As can be seen from the above table, the classification model Random Forest used in this article has good performance on the above four data sets. The accuracy and the F1 value of the comprehensive index reflecting the precision rate and recall rate are higher than those of the other three classification models.

The above experiments prove that the method proposed in this paper can effectively improve the performance of the classifier on the WHL data set. In order to better verify the applicability of the model, we compare the method MSMOTE-GAN proposed in this paper with the classic Bootstrap and tableGAN on SPECTF, WHL, and Page Blocks data sets, and the Random Forest model with better performance in the above experimental process is used as the classifier, and the result is shown in Table 8.

It can be seen from Table 8 that MSMOTE-GAN can improve the performance of the classifier on both the WHL and SPECTF data sets. But the effect of MSMOTE-GAN, tableGAN, and Bootstrap models on the Page Blocks data set is not as good as that of Origin data. The experimental results show that the MSMOTE-GAN model proposed in this paper can improve the classification performance of classifier on the moderate class imbalanced data set, and the AUC value is significantly improved. But for data sets with extremely imbalanced samples (IR > 46.6), the processing ability is poor, and the AUC value is reduced. In summary, the MSMOTE-GAN model is suitable for data environments, where the overall sample size is small, and the sample imbalance rate is moderate. Except this, it can be used not only in the manufacturing field, but also in the medical field where data attributes are related. It proves that the model has a certain versatility.

## 5. Conclusions

Aiming at the problem of product quality classification in a small sample data and imbalanced data environment, this paper proposes a data generation model that combines MSMOTE and GAN to solve the problem. In the data generation model, MSMOTE is used to balance the positive and negative samples in the data. GAN generates fake data with the same distribution as the original data based on a balanced data set and expands the sample size to solve the problems of overfitting and insufficient model expression ability that occur when the sample size is too small. The Random Forest algorithm is used for subsequent quality classification. Based on the MSMOTE-GAN data generation model and Random Forest, the quality classification framework for water heater liner is proposed. The proposed method is verified by using the actual data (WHL) coming from the factory production line and public data set (SPECTF and Page Blocks). The data generation model can effectively improve the performance of the classification model. Experiments show that MSMOTE-GAN has better performance than Bootstrap and tableGAN on moderate class imbalanced data set, and the performance on extremely imbalanced data sets needs to be improved. In addition, our comparative experiments on the SPECTF medical data set prove that the MSMOTE-GAN data generation model proposed in this paper can also be applied to the medical industry, manufacturing, and other fields facing small sample data and imbalanced data environment and has a certain versatility.

## Figures and Tables

**Table 1:** Parameters affecting the quality of water heater liner.

Parameter 1	Predegreasing temperature
Parameter 2	Predegreasing pressure
Parameter 3	Predegreasing concentration
Parameter 4	Degreasing temperature
Parameter 5	Degreasing pressure
Parameter 6	Degreasing concentration
Parameter 7	Silane pressure
Parameter 8	Silane PH value
Parameter 9	Activation point of silane
Parameter 10	Pressure of washing I
Parameter 11	Pressure of hot water washing
Parameter 12	Pressure of washing II
Parameter 13	Conductivity of washing II
Parameter 14	Pressure of washing III
Parameter 15	Conductivity of washing III
Parameter 16	Pressure of washing IV
Parameter 17	Conductivity of washing IV
Parameter 18	Pressure of pure washing
Parameter 19	Conductivity of pure washing
Parameter 20	Oven temperature
Parameter 21	Curing furnace temperature

**Table 2:** Data set details.

	SPECTF		WHL		Page Blocks	
	Positive	Negative	Positive	Negative	Positive	Negative
Train set	145	41	697	96	3757	74
Test set	67	14	303	37	1601	41
Total	212	55	1000	133	5358	115
IR	3.9		7.5		46.6	

**Table 3:** Confusion matrix structure.

Confusion matrix		Predict class	
		Positive	Negative
Actual class	Positive	TP	FN
	Negative	FP	TN

**Table 4:** Model training parameters.

*K*	batch_size (m)	Epoch	learning_rate	beta_1
5	64	200	0.0002	0.5

**Table 5:** Confusion matrix of Random Forest.

Confusion matrix of random forest		Predict class	
		Positive	Negative
Actual class	Positive	303	0
	Negative	37	0

**Table 6:** Accuracy of four classifiers.

Models	DT	RF	MLP	XGBoost
Accuracy	0.891	0.891	0.891	0.891

**Table 7:** Performance metrics based on balanced data (different data process models).

Data process models	Models	Accuracy	Precision	Recall	F1
SMOTE	DT	0.823	0.941	0.855	0.896
	RF	0.911	0.939	0.964	0.951
	MLP	0.806	0.907	0.871	0.889
	XGBoost	0.838	0.943	0.871	0.906

MSMOTE	DT	0.823	0.941	0.855	0.896
	RF	0.911	0.939	0.964	0.951
	MLP	0.806	0.907	0.871	0.889
	XGBoost	0.838	0.943	0.871	0.906

MSMOTE-GAN	DT	0.897	0.950	0.934	0.942
	RF	0.944	0.952	0.987	0.969
	MLP	0.876	0.964	0.894	0.928
	XGBoost	0.941	0.949	0.986	0.968

**Table 8:** Accuracy and AUC value on SPECTF, WHL and Page Blocks data sets.

Models	SPECTF (IR = 3.9)		WHL (IR = 7.5)		Page Blocks (IR = 46.6)	
	Accuracy	AUC	Accuracy	AUC	Accuracy	AUC
Origin data	0.827	0.839	0.891	0.560	0.984	0.987
Bootstrap	0.815	0.843	0.891	0.618	0.985	0.984
tableGAN	0.827	0.886	0.893	0.633	0.979	0.983
MSMOTE-GAN	0.840	0.898	0.944	0.850	0.970	0.980

## Data Availability

The data used to support the findings of this study have not been made available because the WHL dataset is from actual production lines and involve corporate confidentiality.

## Nomenclature

ADASYN: Adaptive synthetic
AE: Auto-encoder
AUC: Area under curve
CTGAN: Conditional tabular generative adversarial network
DT: Decision tree
FN: False negative
FP: False positive
FPR: False positive rate
GAN: Generative adversarial network
KNN: K-nearest neighbor
MLP: Multi-layer perceptron
MSMOTE: Mahalanobis synthetic minority oversampling technology
RF: Random forest
ROC: Receiver operating characteristic curve
SMOTE: Synthetic minority oversampling technology
TGAN: Tabular generative adversarial network
TN: True negative
TP: True positive
TPR: True positive rate
WHL: Water heater liner data set.
